# Sample and Circular Advertising—Traveling Representatives

**Published:** 1905-02

**Authors:** 


### EDITORIAL NOTES AND COMMENTS.

The Business office of THE JOURNAL-RECORD is No. 1007-1008 Century Building.

The Editorial office is 318-319-320 The Prudential.

Address all Business communications to Dr. M. B. Hutchins, Mgr.

Make remittances payable to THE ATLANTA JOURNAL-RECORD OF MEDICINE.

On matters pertaining to the Editorial and Original Communications address Dr. Bernard Wolff, Atlanta.

Reprints of original articles will be furnished at cost price. Requests for the same should always be made *on the manuscript*.

We will present, post-paid, on request, to each contributor of an original article, twenty (20) marked copies of THE JOURNAL-RECORD containing such article.

### SAMPLE AND CIRCULAR ADVERTISING—TRAVELING REPRESENTATIVES.

SPEAKING wholly from the standpoint of the busy city doctor, who does not furnish his patients with medicine, we can state it as a fact that the mass of samples sent to, or left with, us are a dead loss and waste for the manufacturer. Samples are hastily set down in any available place and forgotten, as they multiply and become covered with dust. Often the doctor never sees them again, unless perchance he looks them over to see if he can save a little drug-store expense to a poor patient. Finding something which "may suit," he gives it to the patient.

We have heard of a type of commercial doctor who, unable to get fees from poor patients, sells them samples. We know of one doctor who needed the space occupied by an accumulation of samples and proceeded to sell the lot at wholesale to a druggist. In the majority of cases, however, the conglomerate mass is thrown away.

If they are kept, they often become an eyesore alike to physicians and patients, yet, covered with dust, give the impression of dead and useless stuff.

We believe a plan more fruitful would be to send, from time to time, a transferable order to the doctors directing some druggist to deliver a sample bottle or package—such samples being furnished the druggist for this purpose.

This would obviate much of the trouble and useless expense above detailed. Medical journals carrying advertisements could, also, state the method described, thus familiarizing the doctors with the plan as well as conveying to them the welcome news that their offices would no longer be a storehouse of either excellent, good, bad, or worthless medicines.

Representatives of manufacturers of drugs, medicines and chemicals often bear the title of "M.D." Most of them are bright and rather agreeable men. Some of them have missed their calling and should be ranked with the most troublesome book agent. They forget that doctors' patients often pay, and pay well, for an interview. The patient has to have advice, and it is the doctor's business to supply it. For this he receives compensation. The drug man, often representing a house which "never advertises in medical journals," gives the doctor advice and literature, physiological, pathological, chemical, unsought, usually undesirable.

He is not competent to teach, he has no claim on the doctor's time, he has no right to come uninvited to add to the wear and tear of the furnishings, the work of the janitors, the worries of a man sufficiently burdened with the ills of his patients and their haste to be served.

Advertisers who have persisted for years in using medical journal mediums now find their products standard and kept in every first-class drug-store. This method gives no annoyance nor offense, is the least of all wasteful, is legitimate.

Circulars, blotters, etc., come last and least. One-cent mail typifies rapid transit in its *precipitated* flight to the waste-basket. Nice looking letters or imitations of invitations soon become recognizable and share the fate of the "one-centers."

The waste-paper man derives much of his revenue from circular matter thrown away. We regret to see, at times, symptoms of attacks of this form of advertising among some of our one-time friends.

We do not believe any of it pays as well as legitimate advertising in honest medical journals.

